# HiChIA-Rep quantifies the similarity between enrichment-based chromatin interactions datasets

**DOI:** 10.64898/2026.02.01.703086

**Published:** 2026-02-03

**Authors:** Sion Kim, Joseph T. Jackson, Henry B. Zhang, Minji Kim

**Affiliations:** 1 Gilbert S. Omenn Department of Computational Medicine and Bioinformatics, University of Michigan, Ann Arbor, MI 48109, USA.; 2 Department of Electrical and Computer Engineering, University of Michigan, Ann Arbor, MI 48109, USA.; 3 AthenaHealth, Boston, MA 02135, USA.

**Keywords:** 3D genome biology, ChIA-PET, HiChIP, ChIATAC, HiCAR, computational biology, graph signal processing, software tool

## Abstract

3D genome mapping technologies ChIA-PET, HiChIP, PLAC-seq, HiCAR, and ChIATAC yield pairwise contacts and a one-dimensional signal indicating protein binding or chromatin accessibility. However, a lack of computational tools to quantify the reproducibility of these enrichment-based 3C data prevents rigorous data quality assessment and interpretation. We developed HiChIA-Rep, an algorithm incorporating both 1D and 2D signals to measure similarity via graph signal processing methods. HiChIA-Rep can distinguish biological replicates from non-replicates, cell lines, and protein factors, outperforming tools designed for Hi-C data. With a large amount of multi-ome datasets being generated, HiChIA-Rep will likely be a fundamental tool for the 3D genomics community.

## BACKGROUND

The three-dimensional (3D) genome organization is recognized as an important aspect of gene regulation and cellular functions. To probe physical proximity of the genome, researchers developed several technologies based on imaging and high-throughput sequencing. Among the widely used sequencing-based methods are Hi-C (Lieberman-Aiden et al., 2009) and ChIA-PET (Fullwood et al., 2009), which rely on proximity ligation to link two—and only two—genomic loci that are physically close but potentially far apart along the DNA sequence. A key difference is that ChIA-PET encompasses the chromatin immunoprecipitation (ChIP) step to obtain chromatin interactions mediated by a specific protein factor of interest. The original ChIA-PET protocol has been improved by incorporating *in situ* digestion as described in (Rao et al., 2014), with minor variations among HiChIP (Mumbach et al., 2016), PLAC-seq (Fang et al., 2016), and *in situ* ChIA-PET (Wang et al., 2021) protocols. More recently, methods utilizing both Tn5 transposase and chromatin proximity ligation were effective in capturing chromatin contacts among open chromatin regions. For example, Trac-looping (Lai et al., 2018), HiCAR (Wei et al., 2022), and ChIATAC (Chai et al., 2023) experiments enrich for chromatin interactions among accessible chromatin sites. All of these and related techniques yield two types of information: (1) pairwise contacts as in Hi-C experiments (chromatin loops), and (2) one-dimensional signal intensity tracks as in ChIP-seq or ATAC-seq data (enrichment coverage). Note that the ‘chromatin loops’ in these assays refer to the pairwise contacts after processing the data with ChIA-PIPE (Lee et al., 2020) or other pipelines, and are not constrained to the ‘dots’ in the Hi-C contact maps. A commonality among ‘enrichment-based 3C assays’ is the two data modalities that are generated – the chromatin structure and enrichment coverage – and we will sometimes refer to such assays, collectively, as ‘ChIA-PET’.

By leveraging on these experimental techniques, researchers discovered that our genome is tightly organized into compartments and domains (Hi-C) and that gene regulation is aided by physical contacts between enhancers and promoters (ChIA-PET, ChIATAC). In addition to the independent labs, the ENCODE (ENCODE project consortium, 2004) and 4D Nucleome consortia (Dekker et al., 2017; Reiff et al., 2022; Dekker et al., 2025) have generated a large number of ChIA-PET datasets—specifically, 2 replicates each of CTCF (CCCTC-binding factor) and RNAPII (RNA Polymerase II) ChIA-PET data spanning more than 20 cell types. Given the significance of profiling accessible chromatin sites in understanding the regulatory roles of potential enhancers and promoters on gene transcription, there will likely be a plethora of Trac-looping, HiCAR, ChIATAC, and their derivatives jointly profiling open regions and chromatin contacts. However, the field currently lacks robust computational tools to analyze these enrichment-based 3C datasets.

One of the critical tasks is to assess the quality of ChIA-PET data by comparing two replicates, since researchers often rely on this information to pool multiple replicates for downstream analyses. Unfortunately, due to the lack of computational methods, the current practice relies on qualitative visual inspections of the loops and coverage tracks, which can result in highly variable and inconsistent assessments. Alternatively, one may convert the loops into a 2D contact map and apply the tools that are designed to measure similarity between two Hi-C replicates. Representative software packages include GenomeDISCO (Ursu et al., 2018), HiCRep (Yang et al., 2017), HiC-Spector (Yan et al., 2017), and QuASAR (Sauria and Taylor, 2017). The authors of these tools have demonstrated that using similarity measures such as stratum-adjusted coefficient of the contact map and graph smoothing outperforms simple Pearson or Spearman correlation coefficients in distinguishing replicates and non-replicates. However, our results indicate that these tools are not applicable to ChIA-PET data as they often assign similar or higher score to non-replicates than to replicates. More importantly, these existing methods do not utilize the one-dimensional enrichment coverage inherent in the enrichment-based 3C data that are critical indicators of protein binding or open chromatin sites. Two methods were proposed to overcome this limitation. HPRep (Rosen et al., 2021) extended HiCRep by modeling binding intensities while AREDCI (Sun et al., 2024) proposed a self-similarity-based algorithm. However, their utilities were limited to a particular experimental type and file format, often requiring users to process the data from raw FASTQ file using specific pipelines tailored for the experimental protocol (e.g., restriction enzymes for HiChIP and PLAC-seq; linker sequence for ChIA-PET). Therefore, 3D genomics field is still missing a sophisticated algorithm implemented in a user-friendly software tool to compare any datasets from enrichment-based 3C assays.

To fill in this gap, we developed HiChIA-Rep—high-resolution chromatin interaction analysis via reproducibility assessment—a Python package for quantifying the similarity between two ChIA-PET or related datasets. Our idea is rooted in graph signal processing, where we convert pairwise chromatin loops into a graph: each node represents a genomic region and an edge between two nodes represent a ChIA-PET loop between the two genomic loci (i.e., anchors). We utilize the enrichment information intrinsic to ChIA-PET experiments by normalizing the contact maps with the enrichment coverage tracks to account for the non-uniform coverage whereas Hi-C normalization methods assume uniform coverage across the genome. We then simulate how the enrichment signal disperses on the chromatin graph via random walk, thereby blending information from both the enrichment signal and the chromatin structure. When tested on ChIA-PET, HiChIP, PLAC-seq, ChIATAC, HiCAR data in human, Drosophila melanogaster, and mouse cells, HiChIA-Rep outperformed existing methods in terms of both the accuracy and runtime. In particular, HiChIA-Rep can: (1) clearly distinguish biological replicates from non-replicates, (2) quantify biological differences between ChIA-PET libraries of variable conditions (e.g., wild type vs. treated), (3) identify sub-regions of the genome that are different or similar, 4) compute the pairwise similarities of multiple cell lines to construct the chromatin interaction phylogeny that is representative of developmental trajectories.

## RESULTS

### Overview of HiChIA-Rep

**Figure 1:**
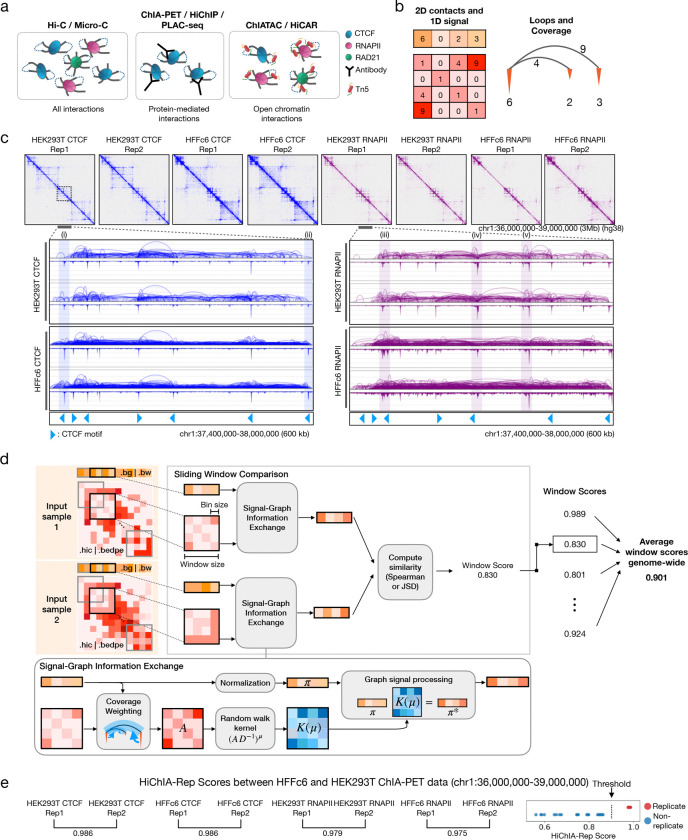
Overview of enrichment-based 3C technologies and HiChIA-Rep method. **(a)** Comparison of 3C assays. Hi-C and Micro-C capture digested and ligated DNA fragments which may be bound to various proteins. Enrichment-based technologies capture a subset of these products that either specifically interact with a protein factor (ChIA-PET, HiChIP and PLAC-seq) or interact with open chromatin regions (ChIATAC and HiCAR). **(b)** Data representations of enrichment-based chromatin interaction data. Left: 1D enrichment signal and 2D contact matrix. Right: Loops (arcs, gray) and coverage (inverted peaks, orange) **(c)** Region chr1:36,000,000–39,000,000 of CTCF and RNAPII ChIA-PET data on HEK293T and HFFc6 human cell lines. Inset shows loops and coverage view and CTCF motifs track (bottom). Blue highlights (i) and (ii) show a peak with different binding signal strength between HEK293T CTCF and HFFc6 CTCF ChIA-PET data, while purple highlights (iii)-(v) show peak with different locations and differential loops between HEK293T RNAPII and HFFc6 RNAPII ChIA-PET data. Each data have two replicates. **(d)** HiChIA-Rep schematics. The program takes two input samples’ enrichment signal (.bigWig or .bedGraph) and contact matrix (.hic or .bedpe) and computes the averaged similarity of a sliding window across the binned genome. The ‘Signal-Graph Information Exchange Module’ (bottom) emphasizes loops with peak support and then performs a *μ*-step random-walk with the kernel *K*(*μ*)=(*AD*^−1^)^*μ*^, where *A* is the adjacency matrix and *D* is the diagonal matrix of the degrees (see Methods). **(e)** Left: HiChIA-Rep window scores (chr1:36–39Mb) using default parameters (*μ* = 5, Spearman similarity) between replicates of HEK293T and HFFc6 ChIA-PET data. Right: Dot plot of HiChIA-Rep window scores (chr1:36–39Mb) between all pairwise combinations of HEK293T and HFFc6 ChIA-PET data. Each dot corresponds to a score comparing either replicates (red) or non-replicates (blue). The threshold is drawn at 0.9.

Hi-C and Micro-C capture all chromatin-chromatin interactions in a population of cells, producing genome-wide unbiased folding structures (Figure 1a). On the same population of cells, ChIA-PET, HiChIP or PLAC-seq captures a subset of the interactions, which are bound to particular proteins such as CTCF, the CCCTC-binding factor (blue). Similarly, ChIATAC or HiCAR captures chromatin-chromatin fragments that interact with open regions. Quantifying the chromatin-chromatin interactions between genomic regions allows one to construct a 2D contact matrix, where the *ij*-th entry corresponds to the number of interactions between binned genomic loci *i* and *j*. ChIA-PET and similar enrichment-based technologies additionally quantify the enrichment level of the protein or open region, allowing one to obtain a vector where each entry denotes the protein binding affinity or DNA accessibility at a particular location along the genome (Figure 1b, ‘2D contacts and 1D signal’). An alternative representation of the data is the ‘loops and coverage’ view, where the enrichment signal is shown as inverted peaks (orange) and the chromatin-chromatin interactions as arcs between the peaks (Figure 1b, ‘Loops and Coverage’).

When comparing two or more ChIA-PET experiments, the data may differ in either the structure, the enrichment coverage, both, or none. For instance, HEK293T and HFFc6 CTCF ChIA-PET contact matrices in a 3 Mb region on chromosome 1 appear to be correlated (Figure 1c), but a closer look into a 600kb subset of the region (inset) shows differences in the CTCF protein binding signals (Figure 1c, i–ii). For RNAPII ChIA-PET data, however, HEK293T and HFFc6 exhibit not only differing binding signals, but also loop structures as shown by (iii)-(v). This noted property motivated us to develop a reproducibility measure that can assess the similarity between two ChIA-PET experiments by incorporating the inherent correlations in the 3D structure and enrichment signals. There are currently limited reproducibility methods for enrichment-based sequencing data, and simple applications of existing methods, such as Hi-C reproducibility metrics, would ignore the enrichment coverage signals. Perhaps a more subtle issue of using Hi-C methods involves the underlying assumption that reads are uniformly sampled across the genome, which is often implicitly assumed by downstream algorithms (e.g. Hi-C normalization methods). By contrast, ChIA-PET data are not expected to have uniform coverage as they enrich for specific subsets of interactions along the genome. As a result, a critical step in ChIA-PET preprocessing is to emphasize interactions (loops) that are supported by strong enrichment signal and to understate others. One computational method is ‘peak support filtering’ that removes loops if the anchors do not coincide with peaks (Figure S1a). The peak support filtered loops remove noise and emphasize relevant biological interactions, such as CTCF loops for CTCF ChIA-PET (top) and enhancer-promoter (E-P) interactions for RNAPII ChIA-PET (bottom). However, this method relies on accurate set of peaks to define regions of high enrichment signal, requiring users to run a peak-calling software and assuming only a binary output (a peak exists or not). Instead, we add the enrichment signals coinciding at the anchors to the respective loop weight, thereby emphasizing strongly supported loops in a continuous, non-binary manner (Figure S1b).

Incorporating this preprocessing technique, we developed HiChIA-Rep, which can quantify the similarity between two datasets from ChIA-PET or related enrichment-based assays (Figure 1d, see Methods for details). Specifically, HiChIA-Rep takes two input samples’ chromatin interactions (.hic or .bedpe) and enrichment signal (.bedGraph or .bigWig), bins the interactions and enrichment signal at a specified bin size, and performs a sliding window across the genome. Each window is fed into the ‘Signal-Graph Information Exchange’ module that is designed to incorporate information from the contact matrix into the enrichment signal, such that when the processed enrichment signals are compared using either the Spearman correlation or Jensen-Shannon Divergence (JSD), the final score reflects discrepancies in both the contact matrix and the enrichment signal. The window scores are then averaged to obtain the genome-wide reproducibility measure between the two input samples. Inspired by the Evoformer in Alphafold2 (Yang et al., 2023), we designed the Signal-Graph Information Exchange module (Figure 1d, bottom) to pass information to and from the chromatin structure and the signals. Specifically, we pass information from the enrichment signal to the contact matrix by coverage weighting (Figure S1b). Then, we view the contact matrix as a weighted adjacency matrix *A* associated with a graph and the enrichment signal to be a continuous measurement on the graph nodes, thereby enabling graph signal processing techniques. We simulate a *μ*-step random walk of the enrichment signal according to the graph structure (Figure 1d ‘Graph Signal Processing’) to allow structural information to be integrated into the enrichment signal. This processed enrichment signal is used to compare the input samples such that information from both data modalities can potentially influence the reproducibility score. The effect of these operations on real data, MCF7 CTCF ChIA-PET rep1, is provided (Figure S1c). Notably, HiChIA-Rep does not rely on machine learning or training steps. Algorithmic details are provided in the Methods section, and the software is publicly available as a Python package.

### HiChIA-Rep outperforms Hi-C reproducibility methods on HEK293T and HFFc6 ChIA-PET data

**Figure 2:**
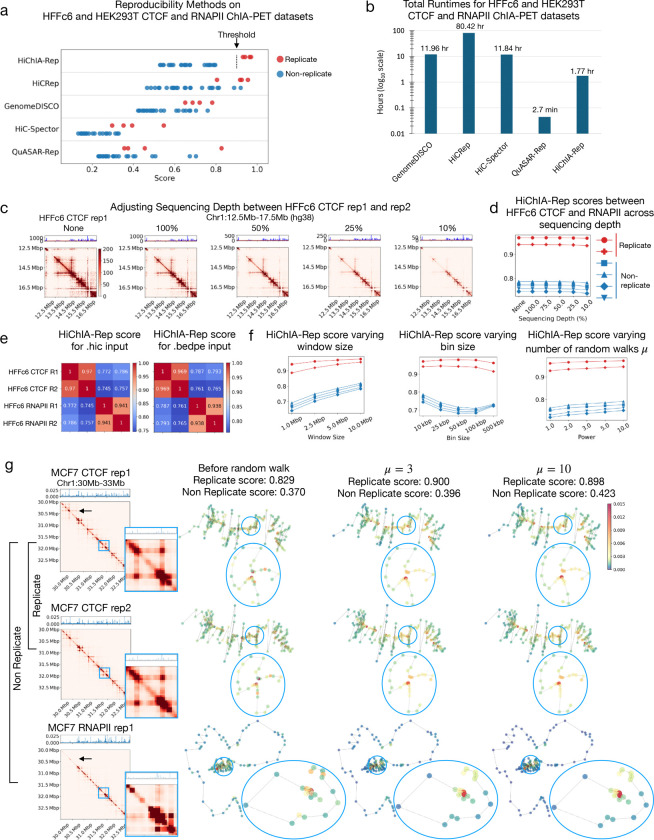
Comparison of HiChIA-Rep against Hi-C reproducibility methods and across sequencing depth and input parameters. **(a)** Dot plot of HiChIA-Rep and Hi-C reproducibility scores between all pairwise combinations of HEK293T and HFFc6 ChIA-PET data. Threshold is drawn at 0.9 for HiChIA-Rep. **(b)** Total runtimes of HiChIA-Rep and Hi-C reproducibility methods on HEK293T and HFFc6 ChIA-PET data. min: minutes; hr: hours. **(c)** 2D contact matrices and 1D enrichment signal of HFFc6 CTCF rep1 (replicate 1) adjusted for sequencing depth with HFFc6 CTCF rep2. Sequencing depth adjustment: no sequencing depth adjustment (None); downsampled such that total number of counts is equal to the smaller total count in a given pair (100%), and further downsampling (75%, 50%, 25% and 10%). **(d)** HiChIA-Rep scores on HFFc6 CTCF and RNAPII ChIA-PET data varying sequencing depth. **(e)** HiChIA-Rep scores on HFFc6 CTCF and RNAPII ChIA-PET data for .hic input (left) and .bedpe input (right). **(f)** HiChIA-Rep scores on HFFc6 CTCF and RNAPII ChIA-PET data varying window size (left), bin size (center), and number of random walks (right) **(g)** Left: Enrichment signal and contact matrix of MCF7 CTCF (rep1, rep2) and RNAPII rep1 ChIA-PET data on chr1:30–33Mb region. Inset (blue border) shows chr1:34,750,000–35,050,000. Right: MDS embedding of contact map with color of points showing enrichment signal before random walk, after random walk (*μ* = 3), after random walk (*μ* = 10). The scores are the Spearman coefficient between the enrichment signals of the chr1:30–33Mb window.

Applying HiChIA-Rep to HEK293T and HFFc6 ChIA-PET data with two replicates each for CTCF and RNAPII, we find consistently high scores (>0.9) between replicates for the 3 Mb region shown in Figure 1c (Figure 1e). We also compared all possible combinations of non-replicate pairs for the same region and show that all non-replicate scores can be separated (<0.9) from the replicate scores (Figure 1e, right). We then sought to establish whether the HiChIA-Rep reproducibility scores can be used to cluster the samples correctly in terms of the cell line and factor. We show a pairwise comparison matrix of genome-wide HiChIA-Rep scores with hierarchical clustering between HEK293T and HFFc6 ChIA-PET data (Figure S1d). Here, HiChIA-Rep correctly clustered based on the factor (RNAPII vs CTCF), followed by the cell line, which aligns with the fact that CTCF binding and loops are conserved across cell lines and species (Klenova et al., 1993; Phillips & Corces, 2009; Moon et al., 2005; Kim et al., 2007; Rudan et al, 2015). Notably, RNAPII should be less conserved than CTCF across cell lines (Tang et al., 2015; Li et al., 2012; Kim et al., 2026), which is reflected in the HiChIA-Rep scores: HEK293T vs HFFc6 RNAPII (0.631–648) vs. HEK293T vs HFFc6 CTCF (0.765–0.796). We then compared our method against four existing Hi-C reproducibility methods, namely HiCRep, GenomeDISCO, HiC-Spector, and QuASAR-Rep (Figure S1e) and found these methods’ clusters to prioritize cell line over protein factor. More importantly, the four methods failed to distinguish replicates from non-replicates, with some non-replicates having higher reproducibility scores than replicates (Figure S1e, Figure 2a). The total run times across all methods on the HFFc6 and HEK293T ChIA-PET dataset (28 pairwise comparisons) are reported: QuASAR-Rep had the shortest run time (2.7 minutes), followed by HiChIA-Rep (106 minutes); GenomeDISCO and HiC-Spector took about 12 hours, while HiCRep was the slowest with ~80 hours (Figure 2b).

### HiChIA-Rep is robust to sequencing depth and parameters

To gauge the effect of sequencing depth on HiChIA-Rep, we used a binomial downsampling scheme to ensure that the number of total counts between two input samples is matched to the lower count of the pair for both the contact map and the enrichment signal (Figure 2c). We generated sub-sampled pairs for HFFc6 CTCF (rep1 and rep2) and RNAPII (rep1 and rep2) ChIA-PET data. Applying HiChIA-Rep on this dataset resulted in scores that remain consistent across sequencing depth ranging from 100 percent to 10 percent (Figure 2d). We then tested the effect of various input types and parameter settings. HiChIA-Rep can take as input either a .hic or .bedpe file for the chromatin interactions, between which results are largely consistent (Figure 2e). This feature is critical to ensure that HiChIA-Rep is a general and versatile tool for users with any standard file format without having to re-process datasets. We then tested the three main parameters of HiChIA-Rep: window size, bin size, and the number of random walks *μ*. We varied window size from 1 Mb to 10 Mb, bin size from 10 kb to 500 kb, and *μ* from 1 to 10. Unless otherwise specified, the other parameters were kept as default parameters specified in the Methods section. The separability of replicates and non-replicates remained stable across the set of parameters (Figure 2f). Interestingly, there appears to be an optimal bin size at around 50 kb or 100 kb (Figure 2f, center), suggesting a tradeoff between noise at small bin sizes and a lack of information at large bin sizes. The total run time for 6 pairwise comparisons took 106 minutes for HiChIA-Rep, which was substantially faster than GenomeDISCO (11.96 hours), HiCRep (80.42 hours), and HiC-Spector (11.84 hours) (Figure 2a). QuASAR-Rep ran the quickest at only 2.7 minutes. We also evaluated whether HiChIA-Rep scores using JSD as the comparison metric was reliable across input types (Figure S2b) and parameters (Figure S2c). As expected, both JSD and Spearman comparison metric had comparable run times. All results across input types and varying parameters ran with peak memory consumption under 30 GB.

To investigate the effect of increasing *μ* on HiChIA-Rep scores, we show a replicate and a non-replicate pair of MCF7 ChIA-PET data in a 3 Mb window on chromosome 1 (Figure 2g). We selected this region as there were major structural differences between CTCF and RNAPII data, as shown in the inset. We plotted a graph, where the nodes’ 2D locations are inferred from multidimensional scaling (MDS) on the contact map and colored the nodes according to the enrichment signal. Focusing on a chromatin loop (inset) that is present in the CTCF data (rows 1 and 2) but not in the RNAPII data (row 3), we find that the random walk operation (*μ* = 3 and *μ* = 10) smooths the signal to nodes adjacent along the linear genome. To further isolate the effect of the random walk on the binding signal, we modified the binding signal to be a delta-like spike for both CTCF and RNAPIl (Figure S2d). Since the signals are identical, the similarity scores before random walk are 1. With random walk (*μ* = 3 and *μ* = 10), however, we find that the signal disperses according to the topology of the chromatin graph: for CTCF at *μ* = 10, the delta spike originally placed on genomic locus A has spread through a chromatin loop and signal has accumulated on loci B. Comparatively, RNAPII does not have this chromatin loop and the signal has not dispersed in the same pattern. As *μ* increases, more information from the contact matrix is being incorporated into the binding signal, specifically *where* to disperse the signal: to nodes that are close in 3D nuclear space (e.g. between the chromatin loop). Indeed, this behavior can be interpreted within the framework of Markov chains, where the random walk progressively incorporates more information from the graph to a point where the signal only depends on the graph (limiting distribution) and not on the initial enrichment signal.

### HiChIA-Rep works on comprehensive ChIA-PET datasets and GM12878 HiCAR data

**Figure 3:**
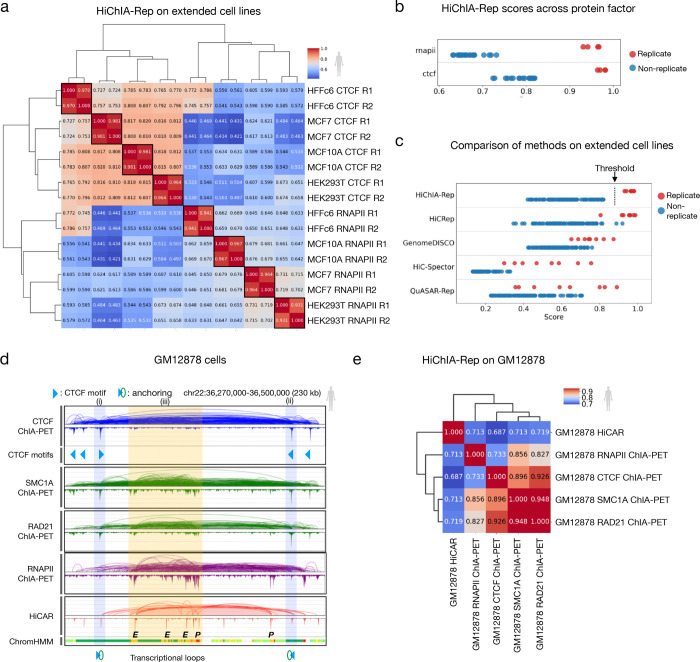
HiChIA-Rep on human ChIA-PET data and GM12878 HiCAR. **(a)** HiChIA-Rep scores between all pairwise combinations of HFFc6, MCF7, MCF10A, HEK293T cell lines for CTCF and RNAPII ChIA-PET (2 replicates each) with hierarchical clustering. R: replicate. **(b)** Dot plot of HiChIA-Rep scores on the same dataset as Figure 3a stratified by protein factors RNAPII and CTCF. For instance, in row 1, red points are replicates between RNAPII samples (e.g., HFFc6 RNAPII R1 vs R2) and blue points are non-replicates between RNAPII samples (e.g., HFFc6 RNAPII R1 vs. MFC10A RNAPII R1). **(c)** Dot plot of HiChIA-Rep and Hi-C reproducibility scores on the same dataset as Figure 3a. The threshold is drawn at 0.9 for HiChIA-Rep. **(d)** Genome browser tracks showing GM12878 data on chr22:36,270,000–36,500,000: CTCF ChIA-PET ‘loops and peaks’ (row 1), CTCF motifs (row 2), SMC1A, RAD21, and RNAPII ChIA-PET (rows 3–5) and HiCAR (row 6), and ChromHMM markings (row 7). TSS (red), gene transcription (dark green), enhancers (light green and yellow). Blue highlight (i) and (ii) shows the anchors of a cohesin-CTCF loop. Orange highlight (iii) shows densely clustered enhancer and promoters. **(e)** HiChIA-Rep scores between all pairwise combinations of GM12878 HiCAR (rep1) and ChIA-PET for RNAPII (rep2), SMC1A (rep1), RAD21 (rep1) and CTCF (rep1) with hierarchical clustering.

We next applied our method on an extended larger ChIA-PET datasets consisting of four human cell lines HFFc6, MCF7, MCF10A, and HEK293T enriched for CTCF and RNAPII with two replicates each (Figure 3a). HiChIA-Rep can distinguish protein factors, cell line and replicates from non-replicates (Figure 3a). Scores are generally higher for CTCF than for RNAPII (Figure 3b), reflecting the conserved nature of CTCF binding across cell lines, whereas RNAPII-associated transcriptional patterns suggest a role that is specific to a cell line. We noticed that HFFc6 CTCF has high correlation with HFFc6 RNAPII (0.745–0.786) as compared to other CTCF-RNAPII similarity scores (0.421–0.674) (Figure 3a), potentially due to the presence of spurious interactions present in the loops. Nevertheless, HiChIA-Rep was the only method that could separate all replicates from non-replicates compared to Hi-C reproducibility methods (Figure 3c). We specifically focused on HiCRep as it is commonly used in assessing the reproducibility of 3C-like data. For the most part, HiCRep replicate scores are higher than non-replicates (Figure S3a). However, HiCRep cannot differentiate between the protein factor CTCF and RNAPII, as shown by the hierarchical clustering (Figure S3a), likely because it is agnostic to the 1-D binding signal differences that are apparent between CTCF and RNAPII. Indeed, the non-replicate scores that are higher than replicates (green boxes) appear between RNAPII and CTCF.

Next, we looked at GM12878 cell line as it has a large profile of genomics data. Here we show ChIA-PET data for CTCF, RNAPII, SMC1A, and RAD21, where SMC1A and RAD21 are subunits of cohesin and therefore can be considered as replicates (Figure 3d). We also show HiCAR data that captures open chromatin associated contacts. In a 230 kb region on chromosome 22, the two subunits of cohesin SMC1A and RAD21 are correlated in both binding and looping (Figure 3d). Highlights (i) and (ii) show two CTCF peaks with convergent motifs and an overarching loop between them, suggesting their role as anchors of a chromatin loop. The bottom two tracks show RNAPII and HiCAR data, which are correlated in region (iii) containing enhancer and promoters as defined by ChromHMM (Ernst & Kellis, 2012), consistent with the fact that open regions tend to be transcriptionally active (Bell et al., 2011). Interestingly, the enhancer-promoter loops are moderately present in the cohesin tracks (RAD21 and SMC1A) but absent in the CTCF, further supporting an emerging notion that cohesin may play a role in some of these enhancer-promoter interactions whereas CTCF is strictly associated with the cohesin-CTCF chromatin looping mechanism (Kim et al., 2026; Guckelberger et al., 2024; Kane et al., 2022). As the authors of HiCAR have noted, some CTCF binding and looping is captured by the HiCAR data, as shown by the blue highlighted region (Wei et al., 2022).

We wanted to test whether the aforementioned relationships among protein factors and open regions could be captured using HiChIA-Rep. Accordingly, HiChIA-Rep has highest correlation between the cohesin tracks SMC1A and RAD21 (0.948) (Figure 3e), which is followed by correlation between cohesin tracks and CTCF (0.896, 0.926). We find that RNAPII is more similar to cohesin (0.856, 0.827) than CTCF (0.733) as expected. HiCAR data appears to be equally similar to RNAPII (0.713) and cohesin (0.713, 0.719), which is plausible as the E-P interactions and cohesin looping are both captured by HiCAR (Figure 3d). We then applied HiChIA-Rep on the biological replicates and found consistent patterns (Figure S3b). Notably, RNAPII ChIA-PET reproducibility is low (0.876), resulting in a non-replicate RAD21 and CTCF ChIA-PET scoring higher (0.926) (green box) than the RNAPII replicates. Nevertheless, HiChIA-Rep accurately clustered the HiCAR replicates, and captured the patterns that RNAPII and CTCF ChIA-PET are the most distant, while cohesin exhibits loops and bindings that resemble both CTCF (forming structural loops) and RNAPII (mediating transcriptional loops).

We then sought out whether HiChIA-Rep could yield consistent results across cell lines and other enrichment-based technologies. Here, we find separability of replicates and non-replicates on HiCAR and ChIA-PET data for GM12878 and H1 cell lines (Figure S3c). We then applied HiChIA-Rep on *Drosophila melanogaster* S2 ChIATAC data, which is a similar enrichment-based method to HiCAR in selecting chromatin contacts associated with open regions (Chai et al., 2022) (Figure S3d). HiChIA-Rep failed to distinguish replicates between RNAPII ChIA-PET, possibly reflecting the poor ChIA-PET data quality as also reported by the authors of ChIATAC (Chai et al., 2022). On human HG00731 HiChIP data, HiChIA-Rep can distinguish replicates from non-replicates (Figure S3e). We then used HiChIA-Rep window scores to select two regions that had low similarity (left) and high similarity between HiChIP CTCF and SMC1 (Figure S3f). With the low similarity region (chr13:85Mb-90Mb) with a score of 0.653, we observed clear structural differences, such as a stripe and additional binding signal in the SMC1 data that is not present in the CTCF. By contrast, in the high similarity region (chr14:60Mb-65Mb) with a score of 0.93, both the structure and binding affinity are highly correlated between CTCF and SMC1. This example suggests that HiChIA-Rep window scores may be used to identify local differences of biological interest, although note that HiChIA-Rep does not provide statistical significance to any differences (see Discussion).

### HiChIA-Rep captures biological patterns in PLAC-seq data on developing mouse embryo tissues

**Figure 4:**
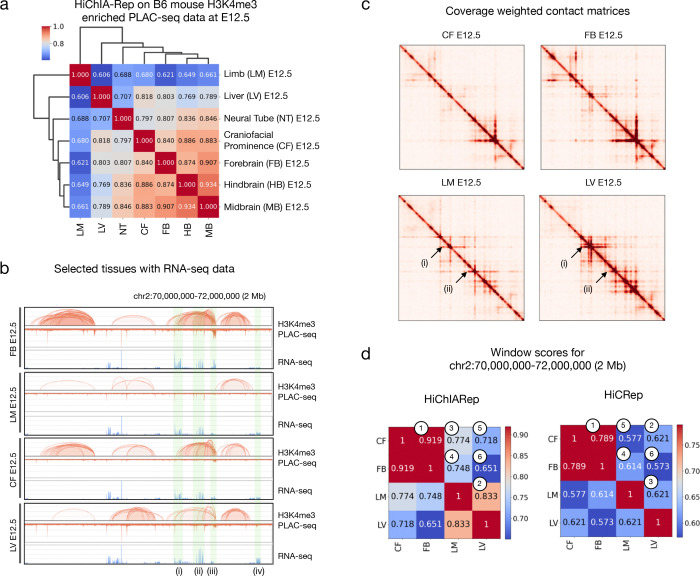
HiChIA-Rep on PLAC-seq data in developing mouse embryo tissue. **(a)** Hierarchical clustering of HiChIA-Rep scores when applied to PLAC-seq data enriched for H3K4me3 on B6 mouse embryo tissues at E12.5 (embryonic day 12.5). **(b)** Genome browser tracks showing mouse embryo tissues on chr2:70,000,000–72,000,000: PLAC-seq ‘loops and peaks’ and RNA-seq for forebrain (FB) (row 1), limb (LM) (row 2), craniofacial prominence (CF) (row 3), and liver (LV) (row 4). The green regions (i)-(iv) highlight differences in RNA-seq between the FB E12.5 and LV E12.5 pair. **(c)** Coverage weighted contact matrices at 10 kb resolution of the same samples shown in panel (b). The arrows (i) and (ii) show shared stripes between limb (LM) E12.5 and liver (LV) E12.5. **(d)** HiChIA-Rep window scores (left) and HiCRep applied to chr2:70,000,000–72,000,000 (right). The number in the circles denotes the ranking from largest to smallest for each respective method.

Another variation of ChIA-PET and HiChIP is PLAC-seq. Enriched for H3K4me3-associated chromatin contacts, publicly available PLAC-seq data (Yu et al., 2025) were generated on developing mouse embryo tissues in time-series from embryonic day 12.5 (E12.5) to postnatal day 0 (P0). We first applied our method by fixing the timepoint to E12.5 to test whether HiChIA-Rep could capture tissue similarities. We find that the brain tissues (midbrain, hindbrain and forebrain) had the highest similarity amongst each other (Figure 4a), whereas limb and liver were most distant to the other tissues. We then looked at a 2 Mb region on chromosome 2 (chr2:70,000,000–72,000,000) for forebrain (FB), craniofacial prominence (CF), limb (LM), and liver (LV) at E12.5 along with RNA-seq data in the corresponding tissues (Figure 4b). While the gene expression profile in this region is relatively similar between tissues, we observed large structural differences in the loop strengths and binding intensities of H3K4me3 PLAC-seq data with high correlation between CF and FB. This pattern can also be seen in the contact matrix view (Figure 4c), where CF-FB have almost identical structures; LM-LV also appear correlated, sharing stripes (i) and (ii). The HiChIA-Rep window scores reflect these correlations (Figure 4d, left), where the CF-FB pair is the highest (0.919) followed by LM-LV (0.833). HiCRep applied to the same window does not follow the same ordering (Figure 4d, right), where the CF-LV pair (0.6213) is more similar than LM-LV (0.6210), which is inconsistent with the structures in the contact map. Interestingly, both HiChIA-Rep and HiCRep ranks the FB-LV pair as the least similar out of all comparisons at 0.651 and 0.573, respectively. This finding was surprising considering that the enrichment coverage signals appear correlated (Figure 4b). Nevertheless, the RNA-seq track shows significant differences (i)-(iv) between LV and FB, more so than for the other pairs, suggesting that such a ranking is consistent with gene transcription for this region. Taken together, HiChIA-Rep can measure discrepancies without solely relying on the enrichment coverage or the contact matrix, providing a relative ordering that is more consistent with local structures than HiCRep.

We then analyzed whether HiChIA-Rep could resolve the similarities between time-points (E12.5-P0) for the forebrain tissue, focusing on the *Neurod6* gene (chr6:55,400,000–56,000,000) involved in the differentiation of the nervous system (Figure S4a). Here, we see a gradual increase in transcription of *Neurod6* across the embryonic days and a slight reduction in the P0 sample. A similar trend is seen with PLAC-seq data, in which structures appear and disappear across time (Figure S4a). This phenomenon is reflected in the HiChIA-Rep scores, where the P0 forebrain sample has a higher similarity to E13.5 or E14.5 than the other time-points (Figure S4b). Interestingly, when we used genome-wide HiChIA-Rep scores and embedded the similarity matrix in 2D coordinates, we find a similar pattern where P0 forebrain tissues cluster close to E13.5 or E15.5, suggesting that the dynamic program of structural changes may not strictly align with tissue labels (Figure S4c). Applying HiChIA-Rep on the entire PLAC-seq dataset, we find that the major differences arise between the tissues more so than the time-points (Figure S4d), which was also apparent in the H3K4me3 ChIP-seq data presented in Yu et al. (2025). The reproducibility of PLAC-seq data also appears to be highly variable for certain samples (e.g. NT E12.5 rep1, LM E12.5 rep1) (Figure S4d).

## DISCUSSION

We described HiChIA-Rep and demonstrated its utility in accurately quantifying the similarity between two datasets generated by a wide range of enrichment-based 3C assays. When tested on ChIA-PET, HiChIP, PLAC-seq, ChIATAC, HiCAR datasets in human, Drosophila melanogaster, and mouse cells, HiChIA-Rep correctly distinguished replicates from non-replicates while the four reproducibility methods designed for Hi-C data could not. We have omitted HPRep and AREDCI due to the practical issue of runtime and memory in preparing the required input file format and the need to use a webserver instead of a command line tool but intend to include them in a thorough benchmark in the future. Through comprehensive simulations in the next round of benchmark, we would like to evaluate the effect of varying the 1D signal while fixing the 2D contacts, and vice versa, with sophisticated methods to model noise and sequencing depth.

Our long-term goal is to understand the relationship between the binding affinity and genome structure and offer ways to control how much information to blend between the two modalities by adjusting the weights of each term accordingly. During the process, we will also learn the biological implications of these experimental protocols. For instance, the enrichment signal may be less prone to noise than 2D contacts for various reasons, such as the increased dimensionality of 2D matrices. Additionally, we expect the 2D contact data to be inherently sparser than the enrichment signal, the reason being that protein binding is required for loop formation: cohesin must be loaded onto the DNA in order for loop extrusion to occur, and similarly CTCF should be bound at the convergent motifs to stabilize this process. In other cases, the enrichment signal may be noisy and uncorrelated with the 2D contacts when cells are not fixed properly during crosslinking, or when some protein factors move faster and spend less time on the DNA than others (e.g., cohesin and RNAPII may be exhibit higher mobility than CTCF, resulting in noisier-looking coverage tracks). The level of random ligation noise also depends on the cell types and experimental conditions.

In terms of testing HiChIA-Rep on simulated data, there are some limitations in the current work. First, the method to adjust for sequencing depth is through a simple binomial distribution and may not be accurate as it is done after the peak support operation. An ideal approach would realistically model the sparsity of both binding intensity and 2D contacts as if the same library were to be sequenced at a lower depth. Second, we did not test the robustness of HiChIA-Rep to noise, such as random ligation, non-specific pull-down during immunoprecipitation, and inefficient transposase activities. In both sequencing depth adjustment and noise simulations, we should also consider the distance-dependency of genome structure, where nearby DNA fragments are more likely to interact by chance than those far apart (Grob et al., 2017).

Finally, the distinction between local and global patterns becomes an important issue in analyzing PLAC-seq data generated from embryo tissues (Figure 4). Certain regions may exhibit a distinct tissue cluster configuration, driven by the activity of transcriptional elements and epigenetic modifications (Figure S4a), yet may not be representative of the “average” chromatin state across the genome (Figure S4d). As a result, local patterns that are meaningful but under-represented may be averaged out and therefore not captured by the (genome-wide) HiChIA-Rep score. Despite this limitation, the current version of HiChIA-Rep outputs window scores, which we used to our advantage in identifying windows with high similarity and low similarity as a means to find local regions with pre-defined patterns (Figure S3f). A better approach to find and quantify the local similarities or discrepancies between samples is to identify statistically differential chromatin contacts considering 1D signal, 2D contacts, variability between replicates, and distance-dependency of structures. We intend to pursue this line of work in the future but is beyond the scope of this manuscript.

## CONCLUSIONS

We present HiChIA-Rep, which has significant advances over existing approaches. Algorithmically, it applies novel graph signal processing method tailored to incorporate both 1D signals and 2D contacts present in enrichment-based 3C assays. Unlike many other tools (Bhattacharyya et al., 2019; Lareau et al., 2018; Rosen et al., 2021) HiChIA-Rep does not require a set of peaks called from 1D signals and instead fully utilizes the tracks as continuous signal without arbitrarily setting a threshold to binarize signal as peaks or non-peaks. Being robust to various parameters is a noted strength. Also, because the kernel operations rely on matrix-vector multiplication, the HiChIA-Rep software package is highly efficient in terms of runtime and may be further improved with the GPU acceleration in the future. Practically, HiChIA-Rep is a versatile, general, and user-friendly Python package. It takes as inputs .bedpe or .hic files for 2D contacts and .bedgraph or .bigwig for 1D signals. As these are the widely used and the standard format adopted by the NIH ENCODE (ENCODE project consortium, 2004) and 4DN consortia (Reiff et al., 2022), users do not need to process the data themselves. Not requiring a peak file is also an advantage as some data processing pipelines may not generate them and peak calling also relies on accurate modeling of restriction enzyme cut sites and other protocol-specific considerations (Lareau et al., 2018).

More broadly, while analyzing PLAC-seq data, we pondered upon the ground truth in developmental trajectory of the cells and whether gene transcriptional landscape should define the cell identity. There is no one-to-one correspondence between genome organization and gene transcription, as exemplified in multiple studies. Rapidly depleting protein factors altered or eliminated chromatin loops, yet transcriptional landscapes remained largely unchanged (Rao et al., 2017; Hsieh et al., 2022; Haarhuis et al., 2017). Studies that inhibited transcription showed little change in genome structure (Li et al., 2018; Jiang et al., 2020). Indeed, genome structure and gene transcription are not perfectly correlated, and understandably so: each modality offers fundamentally different information about the cell, showing distinct slices of common molecular processes in developmental landscapes. Finding how chromatin interactions affect and are affected by gene transcription is important in understanding cell fate decisions, which more broadly will help establish necessary or sufficient sources of information when it comes to defining cell identity. These objectives will likely require careful analyses of multi-modal, multi-omics datasets.

As the 3D genomics field continues to develop methods to jointly profile multiple modalities across a wide range of biological systems, the field will also need sophisticated algorithms and computational frameworks that can holistically view, compare, and reason within the various modalities. There are additional challenges with single-cell multi-ome data, such as ChAIR (Chai et al., 2024), which can jointly profile chromatin structure, open chromatin region, and gene transcription in a single cell; similarly, HiRES (Liu et al., 2023), GAGE-seq (Zhou et al., 2024), LiMCA (Wu et al., 2024), MUSIC (Wen et al., 2024) simultanouesly probe genome folding structure and gene transcription. Indeed, the methods used in HiChIA-Rep can be extended to such data under the computational framework of *structured data* (e.g., graphs and point clouds). We expect HiChIA-Rep to provide important first steps in jointly leveraging chromatin structure and enrichment signals in multi-modal data, enabling algorithmic and biological insights into capturing and understanding the workings of genome structure and cell function.

## METHODS

### 1. Notation

Let the contact matrix of the chromatin interaction frequencies be denoted as $A\in R^{N\times N}$, where ij th entry $A_{i,j}$ denotes the interaction frequency between genomic loci i and j. The genomic loci are binned to a user-defined resolution (see Parameters). Depending on the context, A can refer to the entire chromosomal contact matrix or a specific window (see Sliding Window), which will be made clear in the section. The indices are 1-indexed and ‘:’ denotes a list of numbers (e.g., 1:5 is the list of numbers 1, 2, 3, 4, 5). Let $b\in R^{N}$ denote the corresponding enrichment signal vector, where $b_{i}$ is the enrichment signal at genomic locus i that is binned to the same resolution as the contact matrix. The enrichment signal b may either refer to the binding signal of a protein or DNA accessibility, depending on whether the data is generated from ChIA-PET (also HiChIP, PLAC-seq) or ChIATAC (also HiCAR), respectively. HiChIA-Rep handles both cases identically and the vector b is simply referred to as the ‘enrichment signal’ as a placeholder for both situations. Similarly to the contact matrix, b may refer to the enrichment signal of either the entire chromosome or a specific window.

### 2. Inputs

The main input data to the HiChIA-Rep program is three-fold:

1. Chromatin structure file (either .hic or .bedpe) to construct the contact matrix A.
2. Enrichment signal file (either .bedGraph or .bigWig) to construct the enrichment signal vector b.
3. UCSC chromosome sizes file to know when to stop the sliding window (see Sliding Window).

There are also a few meta-data files, which specify the path locations of the data and also which combinations of the data the user wishes to compare. Please see the Github page (https://github.com/minjikimlab/hichiarep) for how to generate these files. HiChIA-Rep can load in multiple samples at once to compare various combinations of pairs efficiently.

### 3. Parameters

All results in this manuscript are generated using the following default parameter settings with v0.2.2 unless specified otherwise.

The main parameters are the following:

- *window-size* (related to Sliding Window), which is the size in basepairs of the sliding window (default: 5,000,000).
- *bin-size,* which determines the size of the bin in basepairs for the contact matrix A and the enrichment signal b (default: 10,000).
- *window-stride* (related to Sliding Window), which sets the stride factor for the sliding window (default: 2).
- *mu* (referred to as μ in Methods) (related to Signal-Graph Information Exchange) which is the number of random walks to perform (default: 5).
- *Compare method* (related to Computing Similarity), which specifies how to compute the similarity between processed enrichment signals (default: “spearman”).

Additional parameters are the following:

- *chroms_to_load*, which specifies chromosomes to compare (default: “all”).
- *output-dir*, which specifies the results folder name (default: “output”).
- *ba-mult*, which is the value to multiply the enrichment signal values (default: 1)
- *num-cores*, which specifies the number of cores for parallel processing (default: 1)
- *min-hic-value*, which is the minimum Hi-C interaction value to consider as a valid interaction (default: 1)
- *min-bedgraph-value*, which is the minimum enrichment signal value to consider as a valid interaction (default: 1)
- *diagnostic-plots* specifies whether to save diagnostic plots of enrichment signal (default: True)
- *do-output-graph* which specifies whether to output .npy graphs for chr1 windows (default: False).

### 4. Sliding Window

HiChIA-Rep performs a sliding window across the main diagonal of each chromosomal contact matrix $A\in R^{N\times N}$ that is binned at the user-specified resolution (Figure 1d). The motivation for using a sliding window is that entries near the main diagonal contains most of the biologically relevant structures in 3C data, such as TADs, loops, and stripes and is a common assumption for many Hi-C analysis software (e.g., HiCRep). Since enrichment-based assays select for interactions with proteins or open regions, the structures we observe are typically small and focused along the main diagonal, making a sliding window method all the more suitable for enrichment-based data as long as the window size is set appropriately.

Specifically, we extract the values of a square region whose size is specified by parameter *window size*. The first sliding window is positioned such that the top left corner of the window aligns with the top left corner of the chromosomal contact matrix. Subsequent windows slide ‘along the main diagonal’, meaning that they are positioned such that the diagonal elements align with the main diagonal of the chromosomal contact matrix. This will ensure that each sliding window is symmetric and interpretable as a small graph (needed for Signal-Graph Information Exchange). As of the locations along the main diagonal, we slide each window such that there is a 1/(*windowstride*) overlap between adjacent windows (Figure 1d shows the gray and black window for window stride being 2 i.e. ½ overlap). Although adjacent windows may contain the same entries, we implement the window stride feature to ensure that structures which fall between windows are not entirely missed. The sliding window is performed on each chromosome separately until the end of the chromosome, where the last sliding window may end up being smaller than the window size if the chromosome size is not a perfect multiple of the window size. Since many matrix norms and distances are dependent on the dimensions of the matrix, we wanted to ensure that all windows are the same size. To guarantee this, we position the last window such that the lower right corner coincides with the lower right corner of the chromosomal contact matrix (Figure 1d). The sliding window is done similarly for the enrichment signal vector, where we extract entries coinciding with the same genomic regions as the contact matrix (Figure 1d, orange vector).

#### Example

- Chromosome size: 23 Mb.
- Window size: 10 Mb.
- Bin size: 10 kb.
- Window stride: 2.
- The contact matrix A is dimension N×N, where N=2300 (23 Mb / 10 kb = 2300).
- The enrichment signal b is dimension N×1.
- The binned window size is 1000 (10 Mb / 10 kb = 1000).
- Window 1 (genomic location 0–10 Mb)
  - Contact matrix: *A*_1:1000,1:1000_.
  - Enrichment signal: *b*_1:1000_.
- Window 2 (genomic location 5–15 Mb)
  - Contact matrix: *A*_500:1500,500:1500_.
  - Enrichment signal: *b*_500:1500_.
- Window 3 (genomic location 10–20 Mb)
  - Contact matrix: *A*_1000:2000,1000:2000_.
  - Enrichment signal: *b*_1000:2000_.
- Window 4 (genomic location 13–23 Mb)
  - Contact matrix: *A*_1300:2300,1300:2300_.
  - Enrichment signal: *b*_1300:2300_.
- Notably, the last window (window 4) is not smaller than the other windows.

### 5. Signal-Graph Information Exchange

The inputs to this module are a contact matrix and corresponding enrichment signal of an arbitrary sliding window (Figure 1d, bottom). Since this module is independent to the chromosome or the location along the chromosome of the sliding window, for notational simplicity, let the contact matrix be denoted as $A\in R^{M\times M}$ and the corresponding enrichment signal $b\in R^{M}$, where M<N is the number of bins of an arbitrary window.

#### Coverage Weighting

As motivated in the Results, coverage weighting is used to normalize the contact matrices analogous to Hi-C normalization methods. Since all pairwise fragments are captured in a Hi-C or Micro-C experiment (Figure 1a), the resulting contact matrices are expected to have uniform coverage (i.e. their rows and columns should all sum to the same value). Hi-C normalization (e.g., ICE or KR) are computational methods to address the issue where the actual data may not reflect this invariant, which is problematic if certain parts of the genome are under-represented due to GC bias or technical artefacts. Normalization thus serves the purpose of “bringing out” interactions that are artificially under-represented, enabling all downstream algorithms, such as TAD or loop callers to assume that the identified structures are true biological structures, not technical artefacts. ChIA-PET and related enrichment-based technologies require similar preprocessing steps.

A common preprocessing step for ChIA-PET is ‘peak support filtering’, where only loops whose anchors correspond with high enrichment signal are kept (Figure S1a and Results). Operationally, this consists of only keeping interaction $A_{i,j}$ if $b_{i}$ and/or $b_{j}$ is sufficiently high, where “sufficiently high” is determined by a peak-calling software. This simple operation helps bring out relevant interactions whilst reducing noise (Figure S1a). However, the drawbacks include (1) a reliance on peak-calling software and (2) the discrete nature of keeping or filtering interactions based on an arbitrary cutoff threshold, such as determining whether to keep interaction $A_{i,j}$ if $b_{i}$ is high AND $b_{j}$ is high, or alternatively the OR of the conditions. Importantly, we expect to have greater confidence in interactions $A_{i,j}$ where $b_{i}$ is high AND $b_{j}$ is high over interactions $A_{ij}$ where $b_{i}$ is high XOR $b_{j}$ is high. Nevertheless, peak support filtering offers no clear options for how much the former should be emphasized over the latter and is, in general, variable based on the parameters and quality of the peak-calling software.

Our coverage weighting scheme is designed to improve upon ‘peak support filtering’ in all the aforementioned points: to not rely on peak-calling software and to emphasize interactions in a continuous manner, prioritizing interactions where both anchor signals are high over cases where only one anchor signal is high. We achieve this using the continuous enrichment signal value itself. First, we extract the enrichment signals at the loop anchors and then add the values to the existing loop weight (Figure S1b). Mathematically, coverage weighting normalization is,

$${\tilde{A}}_{i,j}=A_{i,j}+b_{i}+b_{j},\;\forall \;i,j\in \{1,\;2,\;\ldots \;,M\}$$

where $\tilde{A}$ is the coverage weighted contact matrix. This ensures that interactions that are well-supported by the enrichment data are emphasized appropriately. At a high level, coverage weighting can be viewed as incorporating information from the enrichment signal vector b into the contact matrix A, reducing noise and technical artefacts in the chromatin contact matrix (Figure S1c). Finally, we perform 2D mean filter blurring on $\tilde{A}$ with fixed filter size of 3 bins, which helps further reduce noise that are particularly present at high resolutions.

#### Random Walk Kernel Construction

The next step is to construct a random walk kernel using the normalized contact matrix from the previous section. For notational simplicity, let the normalized contact matrix be denoted as $A\in R^{M\times M}$. A subtle conceptual point is that the matrix A is viewed as the weighted adjacency matrix associated to a graph with each node corresponding to a binned genomic region. Since A now corresponds to a graph, we can apply graph signal processing techniques. If the enrichment signal represents protein binding, then random walk can simulate how the proteins might randomly disperse on the DNA through time, jumping randomly but preferentially to regions that are close in 3D nuclear space (Figure S2d).

To do this, we first construct a kernel K(μ) that specifies a random walk with μ steps, where μ has the role of the time parameter. Mathematically, the kernel is defined as,

$$K(\mu )={\left(AD^{-1}\right)}^{\mu }$$

where $D=diag\left\{{\left(1^{T}A\right)}_{1},\;\ldots {\left(1^{T}A\right)}_{M}\right\}$ is a diagonal matrix containing the degrees of the nodes and $1\in R^{M}$ is the all-ones vector. Multiplying A with $D^{-1}$ has the effect of normalizing the columns of A to sum to 1, making ${\left(AD^{-1}\right)}^{\mu }$ a μ-step transition matrix associated to a Markov Chain.

#### Normalization

After the kernel K(μ) is constructed, we then normalize the enrichment signal $b\in R^{M}$ such that it sums to 1. Since the enrichment signal values are assumed to be non-negative, the normalized vector is a probability mass function. We define the normalized signal as,

$$\pi_{i}=\frac{1}{\sum_{j}b_{j}}b_{i},\;\forall \;i\in \{1,\;2,\;\ldots \;,M\}$$

where $\pi \in \Delta_{M-1}$ is the normalized enrichment signal vector and $\Delta_{M-1}$ is the (M-1)-dimensional probability simplex. The reasoning behind this step is that we care about the strength of the signal relative to other parts of the vector, rather than the absolute value of the signal. Additionally, if we perform random walk on a probability distribution, then the output is also a probability distribution, enabling us to compare the signals using methods developed from information theory (see Computing Similarity). For brevity, we will refer to π as simply the (normalized) enrichment signal.

#### Graph Signal Processing

The graph signal processing step uses the constructed kernel K(μ) to perform a μ-step random walk of the enrichment signal π. Mathematically, this corresponds to a single matrix-vector multiplication,

$${\left(\pi^{*}\right)}^{T}=\pi^{T}K(\mu )$$

where $\pi^{*}$ is the enrichment signal after performing a μ-step random walk. If π represented protein binding, then $\pi^{*}$ describes how the protein would have dispersed through the graph according to the chromatin structure after each protein takes μ random steps. For an example of this effect, see Figure 2g and Figure S2d.

At a high level, graph signal processing incorporates structural information about the chromatin graph back into the enrichment signal, such that $\pi^{*}$ is a blend of both (1) the original enrichment signal π and (2) chromatin structure from A. We refer to $\pi^{*}$ as the ‘processed enrichment signal.’

### 6. Computing Similarity

Given two processed enrichment signals corresponding a genomic window, one from input sample 1 and another from input sample 2, we compute the window score using either the Spearman correlation or a transformed Jensen Shannon Divergence (JSD) (Lin et al., 2002) (Figure 1d). Whether to use the Spearman correlation or JSD is a user-defined parameter.

Let the processed enrichment signals be $a\in \Delta_{M-1}$ and $b\in \Delta_{M-1}$, where M is the number of bins of an arbitrary window. Assume that the enrichment signals correspond to the ℓth window of the sliding window, where ℓ is an arbitrary number.

The window score $s_{\ell }$ is defined as

$$s_{\ell }=\left\{\begin{array}{rr} spearman(a,b), & Compare\;method\;is\;“spearman”\;\\ 2(1-JSD(a,b))-1, & Compare\;method\;is\;“jsd”\; \end{array}\right.$$

where we simply take the Spearman correlation between the vectors a and b if compare method parameter is “spearman” or a transformed JSD measure if “jsd”. The JSD is the Jensen Shannon Divergence and is

$$JSD(a,b)=0.5D_{KL}(a\;\|\;q)+0.5D_{KL}(b\;\|\;q),$$

where

$$D_{KL}(a\;\|\;b)=\sum_{i=1}^{M}a_{i}{log}_{2}\frac{a_{i}}{b_{i}}$$

is the KL divergence (Kullback & Leibler, 1951) between distributions a and b and

$$q_{i}=0.5a_{i}+0.5b_{i},\;\forall \;i\in \{1,\;2,\;\ldots \;,M\}$$

is the mixture distribution.

The transformation after computing the JSD is to convert the divergence into a similarity score in the range [−1,1], where a high value indicates high similarity.

Let the number of sliding windows across the entire genome be K. Assume that we compute the window score for all windows in the sliding window, obtaining a set of window scores: $\left\{s_{1},s_{2},\;\ldots \;s_{K}\right\}$. The HiChIA-Rep (genome-wide) reproducibility score between input sample 1 and input sample 2 is the average of all the window scores:

$$s_{HiChIA-Rep}=\frac{1}{K}\sum_{\ell =1}^{K}s_{\ell }.\;$$

### 7. Running Hi-C reproducibility methods

We ran Hi-C reproducibility methods HiCRep, GenomeDISCO, HiC-Spector, and QuASAR-Rep using the combined package 3DChromatin_ReplicateQC (v0.0.1) (Yardımcı et al., 2019).

#### HiCRep

We ran HiCRep (Yang et al. 2017) at 50 kb resolution with *maxdist=5000000* (5 Mb) to match the window size for HiChIA-Rep. The smoothing parameter *h=5* based on the recommendations from Yang et al. 2017 (https://github.com/qunhualilab/hicrep).

For the window HiCRep scores (Figure 4d), we used the hicrep python package (v0.2.6), specifically the functions *meanFilterSparse* with *filter size=3* and *sccByDiag* with the entire window range.

#### GenomeDISCO

We ran GenomeDISCO (Ursu et al., 2018) at 50 kb resolution with the following parameter settings: *subsampling*=*lowest, tmin=3, tmax=3, norm=sqrtvc, scoresByStep=no, removeDiag=yes, transition=yes.*

#### HiC-Spector

We ran HiC-Spector (Yan et al., 2017) at 50 kb resolution with *n=20*.

#### QuASAR-Rep

We ran QuASAR-Rep (Sauria et al., 2017) at 50 kb resolution with *rebinning=‘resolution’*.

### 8. Data Processing

#### Converting .bam to .bedgraph

We generated .bedgraph files from .bam files for all the PLAC-seq enrichment signal data (see **Additional File 1**) using samtools (v1.21) (Danecek et al., 2021) and bedtools2 (v2.31.1) (Quinlan et al., 2010). Specifically, we ran *samtools sort*, *samtools rmdup* with option *-s*, and *bedtools genomecov* with options *-ibam* and *-bg*.

#### Converting .pairs to .hic

We generated .hic files from .pairs files for all the PLAC-seq chromatin contacts data (see **Additional File 1**) using JuicerTools (v1.22.01) (Durand et al., 2016) *pre* function with default parameters.

### 9. Analysis

#### Hierarchical Clustering

Hierarchical clustering was performed using *seaborn.clustermap* function with parameters *method="average", metric="euclidean"* from the seaborn package (v0.13.2) (Waskom, 2021).

#### Sequencing Depth Adjustment

We generated subsampled chromatin contact matrices and corresponding enrichment signal for HFFc6 ChIA-PET data (Figure 2c, d) using a binomial sampling scheme. Let p∈[0,1] denote the percentage of further downsampling that we wish to perform (e.g., the p for 100%, 50%, 25% and 10% in Figure 2c is 1.0, 0.5, 0.25, and 0.1, respectively). Given input sample 1 contact matrix $A^{\left(1\right)}\in R^{M\times M}$ and enrichment signal $b^{\left(1\right)}\in R^{M}$ and input sample 2 contact matrix $A^{\left(2\right)}\in R^{M\times M}$ and enrichment signal $b^{\left(2\right)}\in R^{M}$, we perform subsampling twice. The first round of subsampling is to match, in expectation, the higher depth matrix (or vector) to the lower one. The second round of subsampling takes these matrices (or vectors) and then further subsamples both to the specified fraction p of reads.

For the first round of subsampling, we count the total sum of the contact matrices, i.e.,

$$T_{1}=\sum_{ij}A_{i,j}^{\left(1\right)}$$

$$T_{2}=\sum_{ij}A_{i,j}^{\left(2\right)}.$$

If $T_{1}=T_{2}$, then the first round of subsampling can be skipped. Otherwise, assume without loss of generality that $T_{1}>T_{2}$. We subsample the higher depth matrix $A^{\left(1\right)}$ by sampling reads from a Binomial distribution with success probability $T_{2}/T_{1}$,

$${Å}_{i,j}^{\left(1\right)}\sim Binomial\left(A_{i,j}^{\left(1\right)},T_{2}/T_{1}\right),\;\forall \;i,j\in \{1,\;2,\;\ldots \;,M\}$$

and leave the lower depth matrix unchanged,

$${\overset{ˆ}{A}}_{i,j}^{\left(2\right)}=A_{i,j}^{\left(2\right)},\;\forall \;i,j\in \{1,\;2,\;\ldots \;,M\}$$

where ${\overset{ˆ}{A}}^{\left(1\right)}$ and ${\overset{ˆ}{A}}^{\left(2\right)}$ are contact matrices after the first round of subsampling. This procedure is performed similarly for the enrichment signals except for vectors instead of matrices.

The second round performs additional subsampling from a binomial distribution with success probability p, i.e.,

$${\overset{ˆ}{\overset{ˆ}{A}}}_{i,j}^{\left(1\right)}\sim Binomial\left({\overset{ˆ}{A}}_{i,j}^{\left(1\right)},p\right),\;\forall \;i,j\in \{1,\;2,\;\ldots \;,M\}$$

and

$${\overset{ˆ}{\overset{ˆ}{A}}}_{i,j}^{\left(2\right)}\sim Binomial\left({\overset{ˆ}{A}}_{i,j}^{\left(2\right)},p\right),\;\forall \;i,j\in \{1,\;2,\;\ldots \;,M\}$$

where ${\overset{ˆ}{\overset{ˆ}{A}}}^{\left(1\right)}$ and ${\overset{ˆ}{\overset{ˆ}{A}}}^{\left(2\right)}$ are contact matrices after both round of subsampling. This procedure is performed similarly for the enrichment signals.

#### Chromatin Graph Plot

The chromatin graph plots in Figure 2g and Figure S2g were generated by applying non-metric multi-dimensional scaling (MDS) on the contact matrix $A\in R^{M\times M}$. Specifically, we first extract the coverage weighted contact matrix A from the respective samples (see Coverage Weighting). We then compute a matrix of discrepancies Y by

$$Y_{i,j}=\frac{1}{A_{i,j}\;+\;0.1},\;\forall \;i,j\in \{1,\;2,\;\ldots \;,M\}.$$

We then used the shortest-paths distances using the *floyd_warshall_numpy* function from NetworkX (v3.4.2) (Hagberg et al., 2007),

D=floyd_warshall_numpy(Y).

These transformations convert the contact matrix into a matrix of geodesic distances for the MDS algorithm. To obtain the embedding coordinates of each genomic loci, we ran non-metric MDS on D using *sklearn.manifold.MDS* with dimension 2 from the scikit-learn package (v1.7.2) (Pedregosa et al., 2011).

#### MDS on HiChIA-Rep scores

To embed forebrain PLAC-seq samples in 2D coordinates, we used non-metric MDS on the pairwise HiChIA-Rep score matrix. Let the HiChIA-Rep score matrix be denoted as $P^{12x12}$, where we have 12 samples (FB E12.5, E13.5, E14.5, E15.5, E16, P0 with 2 replicates each). We converted the scores into a dissimilarity matrix i.e.,

$$D_{i,j}={\left(1-P_{i,j}\right)}^{2},\;\forall \;i,j\in \{1,\;2,\;\ldots \;,12\}$$

where we applied the square transform to emphasize large distances. Since the scores in P were between 0 and 1, this transformation is monotonic and will preserve the rank order of proximities. We then ran non-metric non-metric MDS on D using *sklearn.manifold.MDS* with dimension 2 from the scikit-learn package (v1.7.2) (Pedregosa et al., 2011).

#### Genome Browser Plot

Tracks were visualized using BASIC Browser (Lee et al., 2020) and contact matrices were visualized using Juicebox Web App (v2.3.6) (Robinson et al., 2018) in Figure S3f.

## Supplementary Material

Supplement 1

Supplement 2

## Data Availability

The datasets supporting the conclusions of this article are included within the article and **Additional File 1**.

## CODE AVAILABILITY

The HiChIA-Rep software is available under the MIT License: https://github.com/minjikimlab/hichiarep.
