# Supplementary material for: HiChIA-Rep quantifies the similarity between enrichment-based chromatin interactions datasets
Source: bioRxiv. 2026 Feb 3:2026.02.01.703086. Preprint. [Version 1] doi: 10.64898/2026.02.01.703086 (PMC12889543; doi:10.64898/2026.02.01.703086)
Supplement: Supplement 2 [file NIHPP2026.02.01.703086v1-supplement-2.pdf]

bioRxiv preprint doi: https://doi.org/10.64898/2026.02.01.703086; this version posted February 3, 2026. The copyright holder for this preprint (which was not certified by peer review) is the author/funder, who has granted bioRxiv a license to display the preprint in perpetuity. It is made available under aCC-BY 4.0 International license.

Figure S1

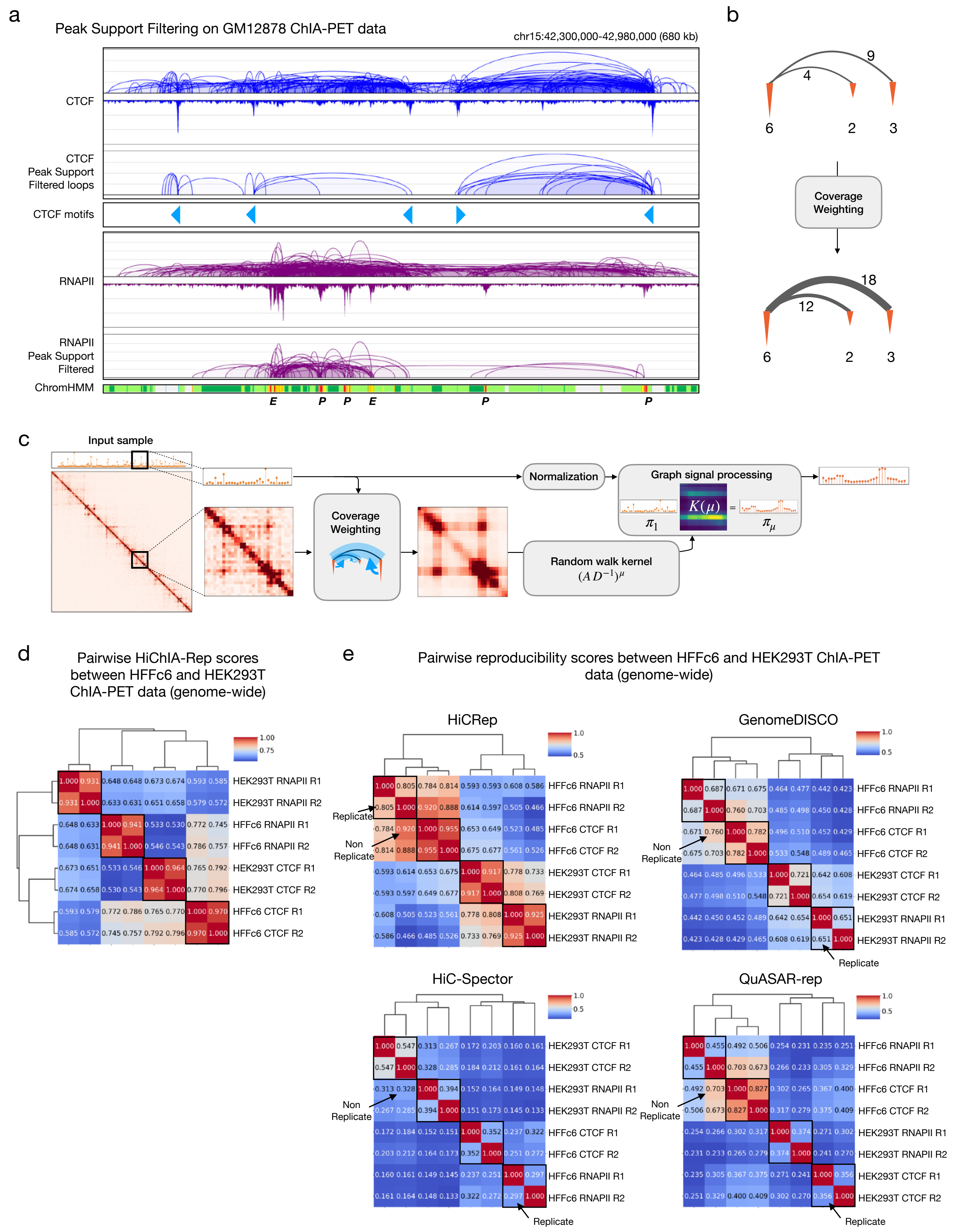

bioRxiv preprint doi: https://doi.org/10.64898/2026.02.01.703086; this version posted February 3, 2026. The copyright holder for this preprint (which was not certified by peer review) is the author/funder, who has granted bioRxiv a license to display the preprint in perpetuity. It is made available under aCC-BY 4.0 International license.

Figure S1: HiChIA-Rep schematics with real example. Related to **Figure 1**.

(a) Genome browser tracks showing GM12878 data on chr15:42,300,000-42,980,000: CTCF ChIA-PET ‘loops and peaks’ (row 1), CTCF ChIA-PET loops after filtering for peak support (row 2), CTCF motifs (row 3), RNAPII ChIA-PET ‘loops and peaks’ (row 4), RNAPII ChIA-PET loops after filtering for peak support (row 5), and ChromHMM chromatin states (row 6). Promoters ‘P’ (red), gene transcription (green), enhancers ‘E’ (yellow). **(b)** Schematic of coverage weighting operation on an example loops and peaks. The loop weight is the sum of the two anchors’ enrichment signal (e.g. 12 = 4 + 6 + 2). **(c)** Signal graph information exchange module on real data: MCF7 CTCF ChIA-PET replicate 1 at chr1:30-33Mb. The inset shows a window chr1:34.75-35.05Mb. The number of random walks is $\mu = 3$. **(d)** HiChIA-Rep scores between all pairwise combinations of HFFc6 and HEK293T ChIA-PET data with hierarchical clustering between samples. **(e)** Hi-C reproducibility scores (HiCRep, GenomeDISCO, HiC-Spector, QuASAR-rep) between all pairwise combinations of HFFc6 and HEK293T ChIA-PET data with hierarchical clustering. Replicate pairs are boxed in black.

bioRxiv preprint doi: https://doi.org/10.64898/2026.02.01.703086; this version posted February 3, 2026. The copyright holder for this preprint (which was not certified by peer review) is the author/funder, who has granted bioRxiv a license to display the preprint in perpetuity. It is made available under aCC-BY 4.0 International license.

Figure S2

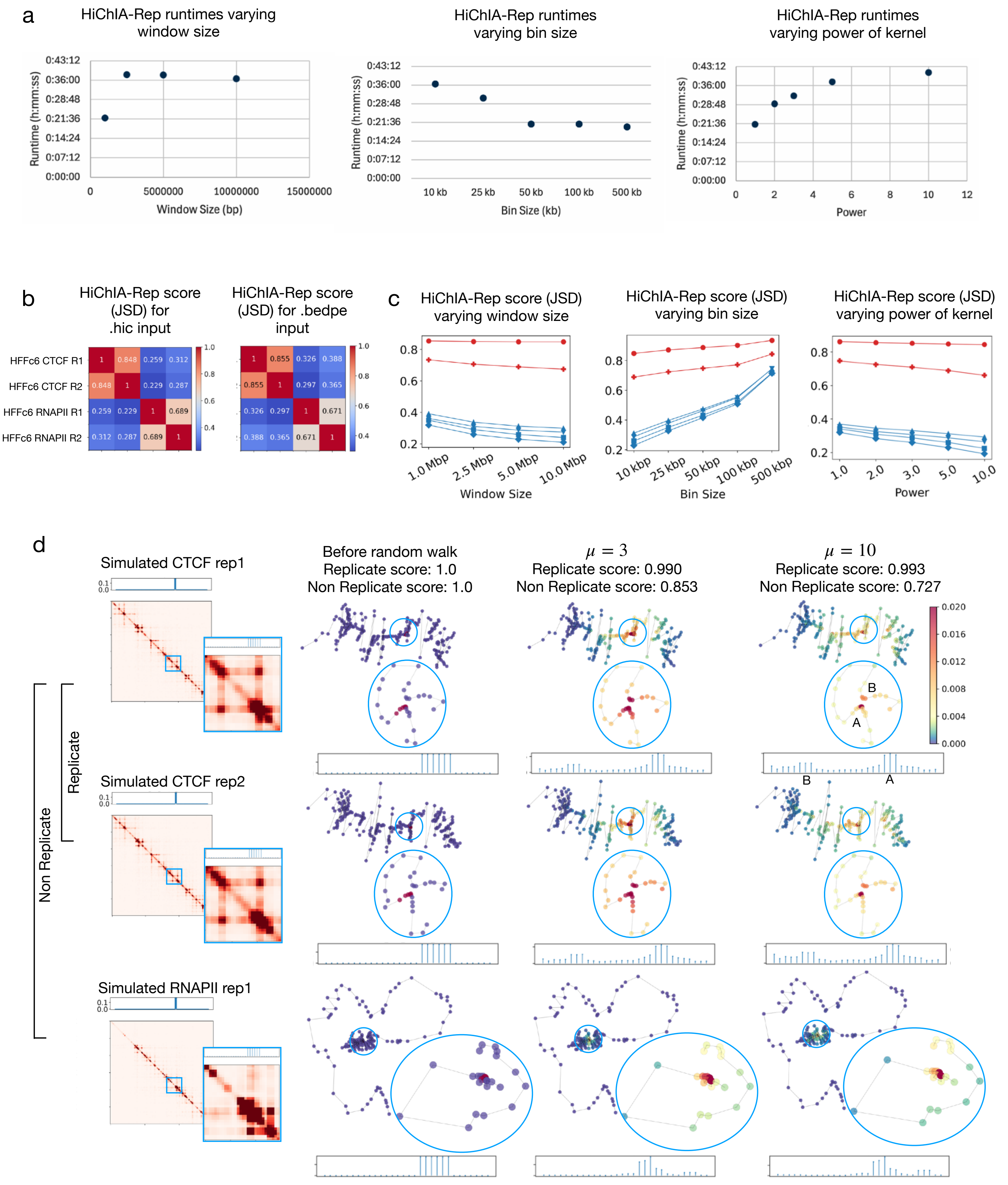

bioRxiv preprint doi: https://doi.org/10.64898/2026.02.01.703086; this version posted February 3, 2026. The copyright holder for this preprint (which was not certified by peer review) is the author/funder, who has granted bioRxiv a license to display the preprint in perpetuity. It is made available under aCC-BY 4.0 International license.

Figure S2: HiChIA-Rep runtimes and effect of different inputs and parameters. Related to **Figure 2**.

(a) HiChIA-Rep total runtimes on HFFc6 CTCF (rep1 and rep2) and RNAPII ChIA-PET (rep1 and rep2) data encompassing a total of 6 pairs (4 choose 2), varying window size (left), bin size (center), and number of random walks (right). **(b)** HiChIA-Rep scores using JSD instead of Spearman coefficient on HFFc6 CTCF and RNAPII ChIA-PET data for .hic input (left) and .bedpe input (right). **(c)** HiChIA-Rep scores using JSD instead of Spearman coefficient on HFFc6 CTCF and RNAPII ChIA-PET data varying window size (left), bin size (center), and number of random walks (right). **(d)** Left: Contact matrix of MCF7 CTCF (rep1, rep2) and RNAPII rep1 ChIA-PET data on chr1:30-33Mb region. The enrichment signal is a delta function placed at chr1:34,920,000-34,980,000. Inset (blue border) shows chr1:34,750,000-35,050,000. Right: MDS embedding of contact map with color of points showing enrichment signal before random walk, after random walk (μ = 3), after random walk (μ = 10). The scores are the Spearman coefficient between the enrichment signals of the chr1:30-33Mb window. The enrichment signals zoomed into the inset region chr1:34,750,000-35,050,000 is shown below each graph. For the enrichment signal shown in μ = 10, the loop anchor regions are A and B.

bioRxiv preprint doi: https://doi.org/10.64898/2026.02.01.703086; this version posted February 3, 2026. The copyright holder for this preprint (which was not certified by peer review) is the author/funder, who has granted bioRxiv a license to display the preprint in perpetuity. It is made available under aCC-BY 4.0 International license.

Figure S3

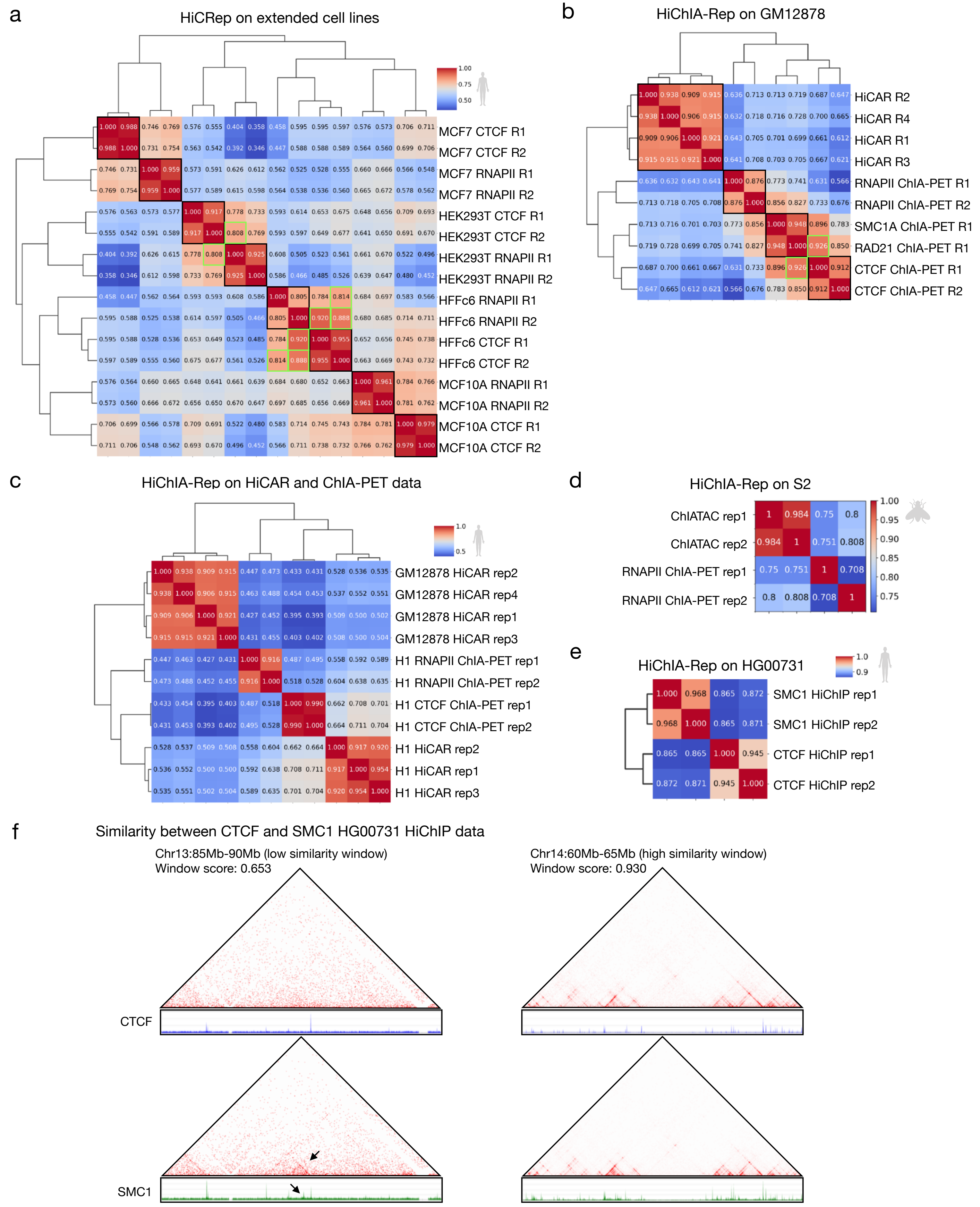

bioRxiv preprint doi: https://doi.org/10.64898/2026.02.01.703086; this version posted February 3, 2026. The copyright holder for this preprint (which was not certified by peer review) is the author/funder, who has granted bioRxiv a license to display the preprint in perpetuity. It is made available under aCC-BY 4.0 International license.

Figure S3: Evaluation of HiChIA-Rep on other cell lines and enrichment-based technologies. Related to **Figure 3**.

(a) HiCRep scores between all pairwise combinations of HFFc6, MCF7, MCF10A, HEK293T cell lines for CTCF and RNAPII ChIA-PET (2 replicates each) with hierarchical clustering. R: replicate. Replicate pairs are boxed in black. Non-replicate scores that are larger than any replicate score is boxed in green. **(b)** HiChIA-Rep on HiCAR (rep1-4) and ChIA-PET enriched for RNAPII (rep1 and rep2), SMC1A (rep1), RAD21 (rep1), and CTCF (rep1 and rep2). Replicate pairs are boxed in black. Non-replicate scores that are greater than any replicate score is boxed in green. **(c)** HiChIA-Rep scores on GM12878 HiCAR (rep1-4), H1 ChIA-PET enriched for RNAPII (rep1 and rep2) and CTCF (rep 1 and rep2), and H1 HiCAR (rep1-3) with hierarchical clustering. **(d)** HiChIA-Rep scores on S2 cell line: ChIATAC (rep1 and rep2) and RNAPII ChIA-PET (rep1 and rep2). **(e)** HiChIA-Rep scores on HG00731 cell line: SMC1 HiChIP (rep1 and rep2) and CTCF HiChIP data (rep1 and rep2). **(f)** HG00731 HiChIP CTCF and SMC1 data (replicate 1) Juicebox and genome browser plots showing the contact matrix and enrichment signal, respectively, of a 5 Mb region with low HiChIA-Rep window score (left) and high HiChIA-Rep window score (right). Juicebox resolution is 10 kb with normalization “None”.

bioRxiv preprint doi: https://doi.org/10.64898/2026.02.01.703086; this version posted February 3, 2026. The copyright holder for this preprint (which was not certified by peer review) is the author/funder, who has granted bioRxiv a license to display the preprint in perpetuity. It is made available under aCC-BY 4.0 International license.

Figure S4

a Forebrain (FB) tissue across time

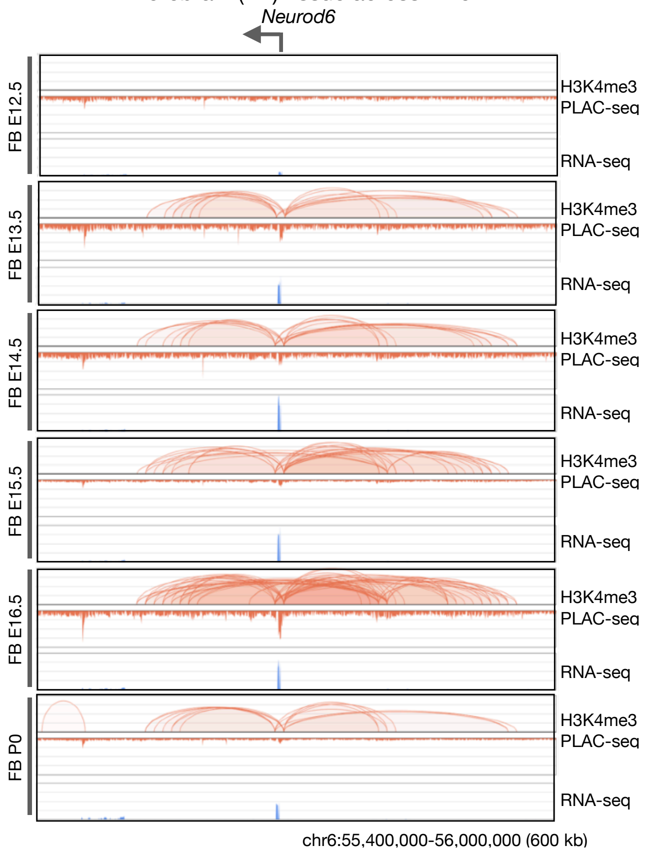

b HiChIA-Rep window scores of forebrain tissue across time

	E12.5	E13.5	E14.5	E15.5	E16.5
P0	0.89	0.94	0.94	0.91	0.86

chr6:52,500,000-57,500,000 (5 Mb)

c MDS plot of HiChIA-Rep on forebrain tissue (genome-wide)

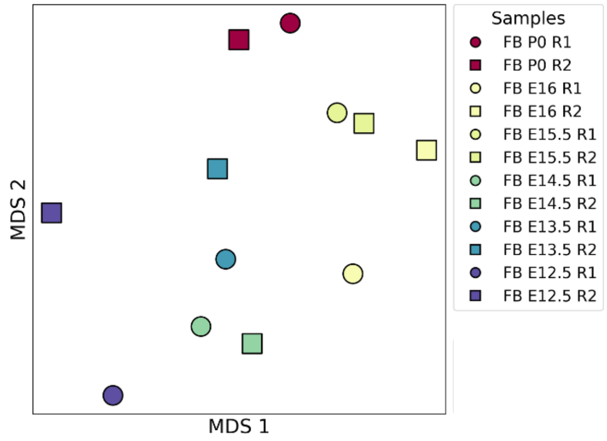

d HiChIA-Rep on B6 mouse H3K4me3 PLAC-seq data

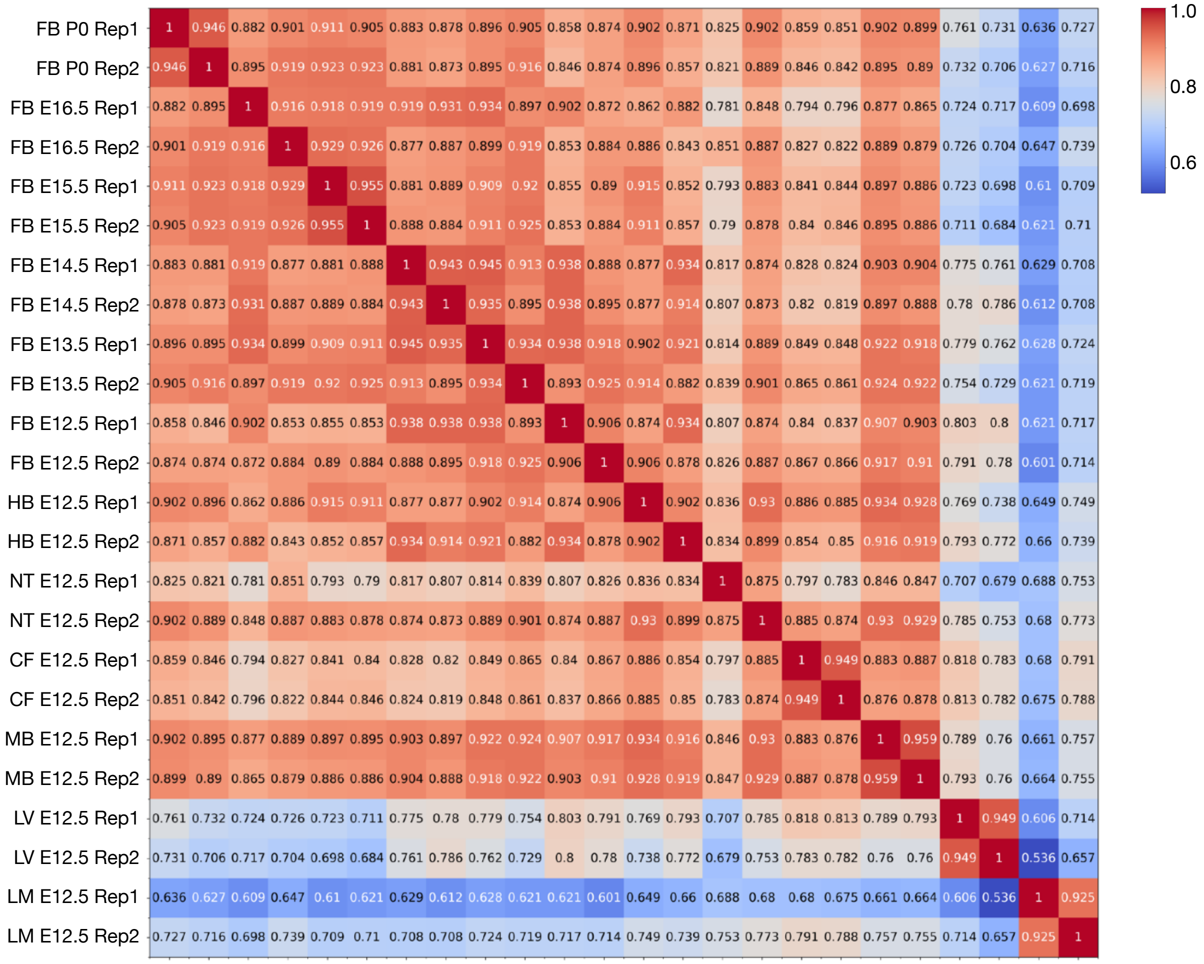

bioRxiv preprint doi: https://doi.org/10.64898/2026.02.01.703086; this version posted February 3, 2026. The copyright holder for this preprint (which was not certified by peer review) is the author/funder, who has granted bioRxiv a license to display the preprint in perpetuity. It is made available under aCC-BY 4.0 International license.

Figure S4: HiChIA-Rep on PLAC-seq data across time. Related to **Figure 4**.

(a) Genome browser tracks on mouse forebrain (FB) tissue (chr6:55,400,000-56,000,000) across time: PLAC-seq 'loops and peaks' and RNA-seq for forebrain tissue at E12.5 (row 1), E13.5 (row2), E14.5 (row 3), E15.5 (row 4), E16.5 (row 5), P0 (row 6). **(b)** HiChIA-Rep window scores of the data presented in panel (a). **(c)** MDS coordinates using genome-wide HiChIA-Rep scores on mouse forebrain tissue. **(d)** HiChIA-Rep scores computed on the entire mouse embryo PLAC-seq dataset, consisting of various tissues (forebrain, hindbrain, neural tube, craniofacial prominence, midbrain, liver, limb) at distinct time points (E12.5-E16.5, P0). FB: forebrain, HB: hindbrain, NT: neural tube, CF: craniofacial prominence, MB: midbrain, LV: liver, LM: limb, Rep: replicate.
